# Activin type I receptor polymorphisms and body composition in older individuals with sarcopenia—Analyses from the LACE randomised controlled trial

**DOI:** 10.1371/journal.pone.0294330

**Published:** 2023-11-14

**Authors:** Tufail Bashir, Marcus Achison, Simon Adamson, Asangaedem Akpan, Terry Aspray, Alison Avenell, Margaret M. Band, Louise A. Burton, Vera Cvoro, Peter T. Donnan, Gordon W. Duncan, Jacob George, Adam L. Gordon, Celia L. Gregson, Adrian Hapca, Cheryl Hume, Thomas A. Jackson, Simon Kerr, Alixe Kilgour, Tahir Masud, Andrew McKenzie, Emma McKenzie, Harnish Patel, Kristina Pilvinyte, Helen C. Roberts, Christos Rossios, Avan A. Sayer, Karen T. Smith, Roy L. Soiza, Claire J. Steves, Allan D. Struthers, Divya Tiwari, Julie Whitney, Miles D. Witham, Paul R. Kemp

**Affiliations:** 1 Cardiovascular and Respiratory Interface Section, National Heart and Lung Institute, Imperial College London, South Kensington Campus, London, United Kingdom; 2 Tayside Clinical Trials Unit (TCTU), Tayside Medical Science Centre (TASC), Ninewells Hospital & Medical School, University of Dundee, Dundee, United Kingdom; 3 Liverpool University Hospitals NHS FT Trust, Clinical Research Network Northwest Coast, University of Liverpool, Liverpool, United Kingdom; 4 AGE Research Group, NIHR Newcastle Biomedical Research Centre, Translational Clinical Research Institute, Cumbria Northumberland Tyne and Wear NHS Foundation Trust and Newcastle upon Tyne Hospitals NHS Trust, Newcastle University, Newcastle upon Tyne, United Kingdom; 5 Health Services Research Unit, University of Aberdeen, Aberdeen, United Kingdom; 6 Medicine for the Elderly, NHS Tayside, Dundee, United Kingdom; 7 Ageing and Health, University of Dundee, Dundee, United Kingdom; 8 Victoria Hospital, Kirkcaldy, United Kingdom; 9 Centre for Clinical Brain Sciences University of Edinburgh, Edinburgh, United Kingdom; 10 Division of Population Health and Genomics, School of Medicine, University of Dundee, Dundee, United Kingdom; 11 Medicine for the Elderly, NHS Lothian, Edinburgh, United Kingdom; 12 Division of Molecular & Clinical Medicine, Dept Clinical Pharmacology, Ninewells Hospital, University of Dundee Medical School, Dundee, United Kingdom; 13 Unit of Injury, Inflammation and Recovery, School of Medicine, University of Nottingham, Nottingham, United Kingdom; 14 NIHR Nottingham Biomedical Research Centre, Department of Medicine for the Elderly, University Hospitals of Derby and Burton NHS Foundation Trust, Derby, United Kingdom; 15 Musculoskeletal Research Unit, Bristol Medical School, University of Bristol, Bristol, United Kingdom; 16 Older Person’s Unit, Royal United Hospital NHS Foundation Trust Bath, Combe Park, Bath, United Kingdom; 17 Institute of Inflammation and Ageing, University of Birmingham, Birmingham, United Kingdom; 18 Department of Older People’s Medicine, Newcastle upon Tyne Hospitals NHS Foundation Trust, Newcastle upon Tyne, United Kingdom; 19 Ageing and Health Research Group, Usher Institute, University of Edinburgh, Edinburgh, United Kingdom; 20 Clinical Gerontology Research Unit, Nottingham University Hospitals NHS Trust, City Hospital Campus, Nottingham, United Kingdom; 21 NIHR Biomedical Research Centre, University of Southampton and University Hospital Southampton NHSFT, Southampton, United Kingdom; 22 Academic Geriatric Medicine, Mailpoint 807 Southampton General Hospital, University of Southampton, Southampton, United Kingdom; 23 Ageing & Clinical Experimental Research (ACER) Group, University of Aberdeen, Aberdeen, United Kingdom; 24 Department of Twin Research and Genetic Epidemiology, King’s College London, Department of Clinical Gerontology, King’s College Hospital, London, United Kingdom; 25 Bournemouth University and Royal Bournemouth Hospital, Bournemouth, United Kingdom; 26 School of Population Health & Environmental Sciences, King’s College London and King’s College Hospital, London, United Kingdom; Cleveland Clinic, UNITED STATES

## Abstract

**Background:**

Ageing is associated with changes in body composition including an overall reduction in muscle mass and a proportionate increase in fat mass. Sarcopenia is characterised by losses in both muscle mass and strength. Body composition and muscle strength are at least in part genetically determined, consequently polymorphisms in pathways important in muscle biology (e.g., the activin/myostatin signalling pathway) are hypothesised to contribute to the development of sarcopenia.

**Methods:**

We compared regional body composition measured by DXA with genotypes for two polymorphisms (rs10783486, minor allele frequency (MAF) = 0.26 and rs2854464, MAF = 0.26) in the activin 1B receptor (*ACVR1B*) determined by PCR in a cross-sectional analysis of DNA from 110 older individuals with sarcopenia from the LACE trial.

**Results:**

Neither muscle mass nor strength showed any significant associations with either genotype in this cohort. Initial analysis of rs10783486 showed that males with the AA/AG genotype were taller than GG males (174±7cm vs 170±5cm, p = 0.023) and had higher arm fat mass, (median higher by 15%, p = 0.008), and leg fat mass (median higher by 14%, p = 0.042). After correcting for height, arm fat mass remained significantly higher (median higher by 4% p_adj_ = 0.024). No associations (adjusted or unadjusted) were seen in females. Similar analysis of the rs2854464 allele showed a similar pattern with the presence of the minor allele (GG/AG) being associated with greater height (GG/AG = 174±7 cm vs AA = 170 ±5cm, p = 0.017) and greater arm fat mass (median higher by 16%, p = 0.023). Again, the difference in arm fat remained after correction for height. No similar associations were seen in females analysed alone.

**Conclusion:**

These data suggest that polymorphic variation in the *ACVR1B* locus could be associated with body composition in older males. The activin/myostatin pathway might offer a novel potential target to prevent fat accumulation in older individuals.

## Introduction

The maintenance of a healthy muscle mass is important in giving individuals the strength to perform the tasks of daily living. Muscle protein is continually turned over such that muscle mass is maintained by balancing the rates of protein synthesis and degradation within relatively tight windows [1]. After the age of approximately 50 in humans there is a gradual decline in muscle mass as the rate of protein degradation exceeds that of protein synthesis (reviewed in [2]). The rate at which the loss of muscle occurs is dependent upon a range of factors including genetics, epigenetics, physical activity, nutrition, and co-morbidities, especially those with a significant inflammatory component [1]. In some individuals the loss of muscle mass and strength can lead to sarcopenia and is sufficient to limit their ability to perform tasks of daily living [3, 4].

Sarcopenia, is defined by the European Working Group on Sarcopenia in Older People (EWGSOP) as the loss of both muscle mass and strength, leading to low muscle mass together with a walking speed less than 0.8m/s or a reduced grip strength [5, 6]. Ultimately, it is the loss of strength that limits the physical capacity of individuals. Loss of muscle mass is a major but not the sole contributor to the loss of strength. Another factor that contributes to the loss of strength is the reduction in “muscle quality” which has been defined in a number of ways including by physiological measurement (e.g. force per unit cross-sectional area [7, 8]) and radiologically (e.g., muscle density on CT or MRI [9]). One factor contributing to a loss of muscle quality is the accumulation of fat in and around the muscle and it has been shown that increased adiposity may increase muscle volume but reduce muscle quality measured by MRI [10]. Furthermore, fat accumulation in muscle is associated with the loss of relative muscle strength [11, 12]. This loss of relative strength is especially true in older women compared to younger women so does not appear to be purely an effect of obesity [13, 14]. Other factors contributing to a loss of strength can include metabolic changes such as mitochondrial dysfunction and insulin resistance [15], such factors may also contribute to fat accumulation. Consequently, like factors that regulate muscle mass itself, factors that govern fat accumulation, mitochondrial function, or insulin regulation, either during development or in response to ageing or environmental factors, will contribute to the loss of muscle quality and thereby strength and physical performance.

Myostatin, perhaps the best-known atrophic signalling protein, signals through a complex of the activin type II B receptor (ACVR2B) and activin type I receptors to initiate a SMAD2/3-dependent signalling cascade. In muscle, this activation increases the expression of the muscle-specific ubiquitin ligases, MuRF and atrogin-1, to promote muscle protein catabolism [16]. Activin A also activates this pathway leading to muscle atrophy [17].

Consistent with a role in the development and maintenance of muscle, polymorphic variation in the activin receptors has been associated with muscle mass and strength. One polymorphism in the first intron of *ACVR2B* (rs2276541) has been shown to associate with lean body mass [18] and haplotype analysis showed an association of a haplotype including the same polymorphism with knee extensor strength in women [19]. However, this variant did not associate with sarcopenia in individuals from the Tibetan plateau [20]. The activin type I receptor *ACVR1B* has also been associated with body composition and strength. Chromosome fine mapping initially identified two polymorphic variations in *ACVR1B* associated with strength [21]. The AA genotype of rs2854464 associated with greater strength than carriers of the minor G allele. This polymorphism has subsequently been found to be over-represented in sprint/power athletes in a cohort of European athletes but not in Brazilian athletes [22]. Located in the 3’-UTR of the *ACVR1B* transcript, this polymorphism is in a putative miR-24 binding site and it is hypothesised that the A allele may have a higher affinity for the miRNA so may lead to reduced *ACVR1B* protein. The second polymorphism, rs10783486 located in intron 1 of the *ACVR1B* gene, also identified by Windelinckx et al [21], has been associated with limb muscle mass measured by ultrasound in older women [23], with individuals with the minor allele having smaller biceps brachii. Furthermore, both polymorphisms have been associated with change in size of the rectus femoris in cardiac patients following exercise training [24], although whether the major or minor alleles were associated with increased response is not clear. However, not all studies show an association of the major allele with increased strength or enrichment in elite athletes. One recent study of the GELAK cohort of Lithuanian athletes and controls shows that the minor rs2854464 allele (G) was enriched in team and power athletes over endurance athletes and controls as well as being enriched in all athlete groups compared to controls [25]. The discrepancy was suggested to be due to study sizes or to dissimilar representation of power sport athletes.

Myostatin is expressed in other tissues including adipose tissue where it has been suggested to contribute to the differentiation of adipocytes [26]. The precise role of myostatin in adipocyte differentiation is controversial with studies indicating both pro- and anti-adipogenic effects of myostatin. In committed adipocyte cell lines (e.g., 3T3-L1 pre-adipocytes) myostatin inhibits cell differentiation [27], whereas in pluripotent stem cells (e.g., CH3 10T1/2, a mesenchymal progenitor) myostatin contributes to differentiation towards an adipogenic phenotype suggesting that it plays a role in the early commitment to the adipocyte cell lineage and away from the myocyte lineage [28, 29]. Consistent with this suggestion, myostatin-null animals have markedly reduced fat mass alongside their increase in muscle mass [30]. Activin also signals via the ACVR2B/ACVR1 complex and similar consequences of activin signalling have been described for muscle breakdown and lipid accumulation.

The above studies suggest that variation in activin receptor function may contribute to body composition in individuals with sarcopenia but a search for studies exploring these polymorphisms in the sarcopenic population did not yield any results. We therefore determined the frequency of the activin type I receptor polymorphisms rs2854464 and rs10783486 in individuals with sarcopenia in the LACE trial and compared these with body mass determined by DXA scans and grip and quadriceps strength at enrolment to determine whether these polymorphisms associated with body composition or strength in individuals with sarcopenia.

## Materials and methods

### Participants and physiological analysis

Participants aged 70 years and over with sarcopenia, according to the EWGSOP definition (2010) [5], were recruited between April 2016 and December 2019 to the LACE trial to investigate inhibition of sarcopenia using leucine and/or ACE inhibition as described (trial registration ISRCTN90094835) [31]. The trial was approved by the East of Scotland NHS research ethics committee (approval 14/ES/1099) and the UK Medicines and Healthcare Regulatory Authority (EudraCT number 2014-003455-61; Clinical Trial Authorisation number 36888/0001/001-0001); the trial was performed in accordance with the ethical standards laid down in the 1964 Declaration of Helsinki and its later amendments. The original study was a double-blind randomised controlled trial and sample preparation and analysis for this work was carried out without any access to information that could identify the individuals involved. The full trial protocol, inclusion and exclusion criteria as well as the main outcomes have been published [31, 32]. Height, weight, and age were recorded, and overall muscle mass was initially estimated by bioimpedance to reduce the total number of DXA scans required as part of the screening process. Individuals with low muscle mass and low muscle strength were recruited to the trial; change in appendicular muscle mass was a key secondary outcome and this was determined by DXA. Baseline data from these appendicular measures (both lean mass and fat mass) were used in this analysis. Maximum grip strength was determined by hand-held Jamar dynamometer and maximum leg strength was determined by measuring isometric voluntary knee extension with a Lafayette 01165 dynamometer (Lafayette Instrument, Lafayette, IN, USA). Measurements were taken with the participant seated, the knee joint at 90 degrees, and with a non-elastic strap running between the chair and the ankle to restrain the dynamometer. Physical performance was determined by measuring 6-minute walk distance (6MW) and short physical performance battery (SPPB). The original study was powered to show difference in SPPB but the study was terminated early due to poor recruitment.

### DNA analysis

Whole blood samples were taken at the first study visit and frozen. DNA was extracted from the first 110 whole blood samples received using the QIAamp DNA blood mini kit according to the manufacturer’s instructions. This set of samples were the first batches received from the various trial sites by the laboratory, and analysis was restricted to this group for purely logistical reasons relating to the COVID pandemic and subsequent funding. DNA genotyping was performed by Taqman qPCR using assays C2022135-10 and C15826314-10 on an ABI 7500 fast thermocycler according to the manufacturer’s instructions. Two samples did not amplify for rs2854464 and 3 samples did not amplify for rs10783486. Data from these individuals were not included in the analysis.

### Statistical analysis

All of the data used in this analysis is available in S1 File. Statistical analysis was performed in Aabel 3.0 with the exception of ANCOVA (SPSS) and the Chi squared test. Due to the low minor allele frequency for both alleles, groups were analysed using a recessive model in which individuals homozygous for the minor allele were combined with heterozygous individuals. Furthermore, given the known sex differences in muscle size and strength and limb fat content all associations with body composition or strength were analysed in males and females separately. Normality was assessed by Shapiro-Wilk test, with differences between groups determined by Mann-Whitney U test for data that did not show a normal distribution and students T-test for normally distributed data. ANCOVA was performed using the General Linear Model in SPSS with height as the co-variate on log_2_ transformed data for limb fat mass. The log_2_ transformation was performed to ensure that the associations between limb fat mass and height in each genotype were parallel. Performing the same analysis on untransformed data did not affect the outcome. Summary statistical output from SPSS is provided in the S2 File.

Whether the alleles were in Hardy-Weinberg equilibrium was determined by Chi squared test using allele frequencies published for the European Caucasian population (rs10783486, MAF = 0.26 and rs2854464, MAF = 0.26) [21].

The threshold for statistical significance (alpha) was taken as 0.05. All available data were used for each comparison and no manipulations were performed to include missing values for individual tests. As this analysis is exploratory no adjustments were made for multiple testing.

#### GTEX data

The Genotype-Tissue Expression (GTEx) Project was supported by the Common Fund of the Office of the Director of the National Institutes of Health, and by NCI, NHGRI, NHLBI, NIDA, NIMH, and NINDS. The data used for the GTEx analyses described in this manuscript were obtained from: the GTEx Portal (https://gtexportal.org/home/) on 24/07/2023.

## Results

The demographics of participants included in this analysis are given in Table 1. All individuals were sarcopenic, as described in the Methods, and the population almost entirely identified as belonging to the White British ethnic group (>99%). In the population as a whole and in both sexes considered separately, the alleles were in Hardy-Weinberg equilibrium. The genotypes and allele frequencies are given in S1 Table. The data analysed are the baseline data from the trial.

**Table 1:** Baseline demographics of the study population.

	female		male	
**n**	56		52	
**Age (years)**	78 (73.5, 84.5)	0.08	77 (74, 81)	0.07
**Weight (kg)**	62.8 (57.4, 71.0)	0.17	81.3 (75.1, 90.35)	0.15
**Height (cm)**	157 ± 6	0.04	171 ± 6	0.04
**BMI (kg/m^2^)**	26.1 (22.9, 28.8)	0.14	27.8 (25.5, 30.6)	0.13
**SARC-F**	4 (3, 5)	0.34	3.5 (3, 4)	0.34
**Total body muscle mass (kg/m^2^)**	5.78 (5.33, 6.03)	0.09	7.34 (6.96, 7.70)	0.08
**Arm fat mass (kg)**	3.20 (2.58, 3.73)	0.32	2.66 (2.06, 3.27)	0.37
**Arm muscle mass (kg)**	3.42 (2.95, 3.81)	0.18	6.04 (5.19, 6.53)	0.15
**Leg fat mass (kg)**	9.24 (7.36, 11.81)	0.32	7.08 (5.85, 8.71)	0.44
**Leg muscle mass (kg)**	10.9 ± 1.64	0.15	16.2 ± 2.33	0.14
**SPPB**	7 (6, 9)	0.36	8 (6, 9)	0.29
**Grip strength (kg)**	13.65 ± 3.40	0.24	24.01 ± 5.34	0.22
**QMVC (kg)**	10.5 (7.6, 14.4)	0.44	15.9 (12.6, 21.4)	0.42

BMI: body mass index, cv: coefficient of variation, SARC-F: Strength, assistance with walking, rising from a chair, climbing stairs, and falls questionnaire score, SPPB: Short Physical Performance Battery score: QMVC: Quadriceps Maximal Voluntary contraction. For normally distributed data values are given as mean ± SD and for data that did not show a normal distribution are median (interquartile range).

Body composition measurements were not available for 1 male. QMVC measurements were not available for 1 female and 6 males.

### rs10783486

**Fig 1:**
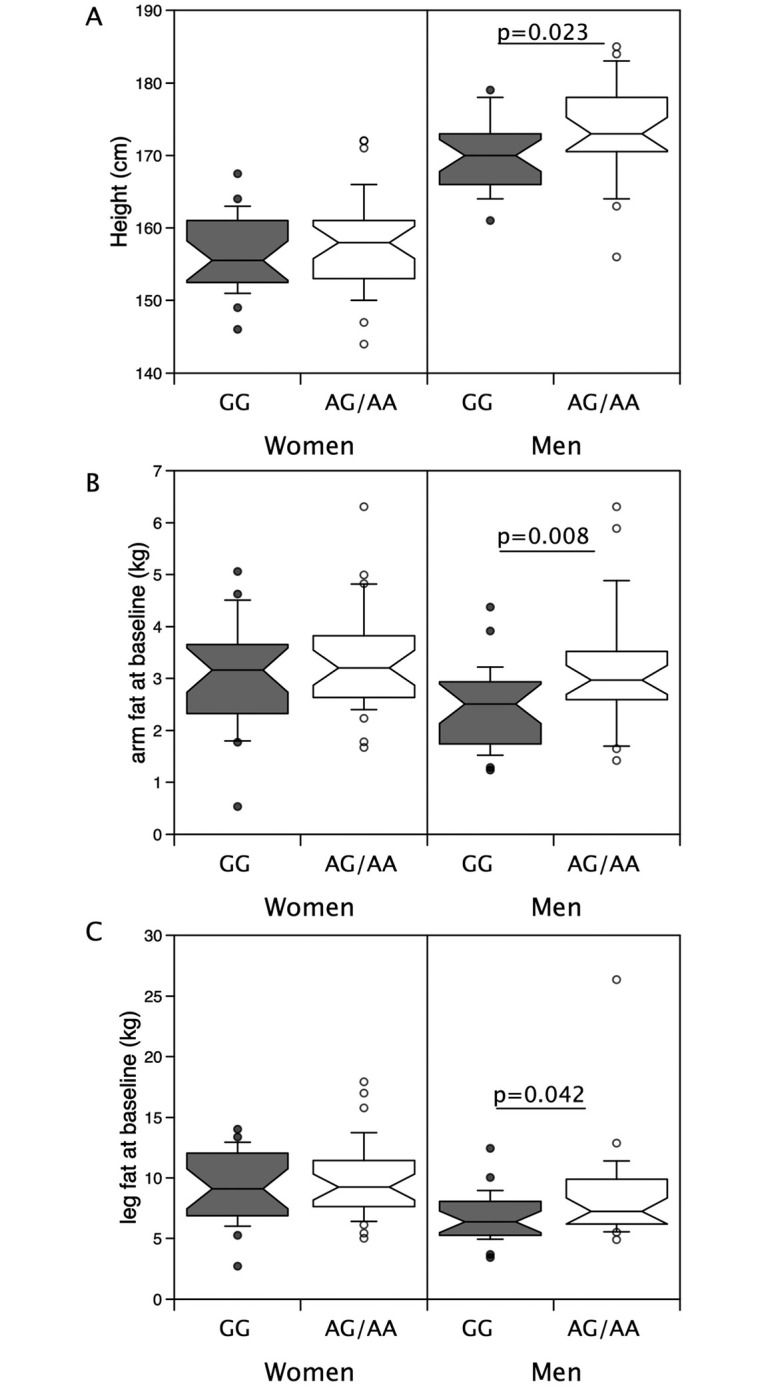
Associations of rs10783846 with height and limb fat mass in individuals in the LACE study. Height and limb fat were compared in males and females possessing the minor allele for rs10783846 with those homozygous for the major allele. Median height (A, p = 0.023), arm fat mass (B, p = 0.008) and leg fat mass (C, p = 0.046) were higher in males with the minor allele than their counterparts homozygous for the major allele. However, in females there were no differences.

Given population differences in body composition and strength between males and females, the sexes were analysed separately. There were no associations of genotype with strength, total body muscle mass (quantified by bioimpedance) or limb muscle mass (quantified by DXA) in either sex (Table 2, S1 Fig). However, males with the rs10783486 AA/AG genotype were taller than GG males (174±7cm vs 170±5cm, p = 0.023) (Fig 1A). They also had higher arm fat mass, (AA/AG median = 2.97kg (IQR 2.60, 3.49), vs GG: 2.51kg (1.76, 2.91) p = 0.008), and leg fat mass (AA/AG 7.25kg (6.29, 9.73) vs GG: 6.37kg (5.27, 7.95), p = 0.042) (Fig 1B and 1C).

**Table 2:** Effect of the minor allele of rs10783846 on body composition and strength in females and males at baseline.

	rs10783486					
	female			male		
	GG	AA/AG	P value	GG	AA/AG	P value
**n**	24	31		25	27	
**Weight**	62.3±11.2	65.8±10.6	0.240	79.5 (73.5, 85.9)	85.3 (77.9, 95.3)	0.059
**BMI (kg/m^2^)**	25.3±3.4	26.5±3.9	0.258	27.5 (25.4, 30.0)	28.3 (25.6, 30.9)	0.273
**Height (m)**	156±5	158±7	0.455	170±5	174±7	**0.023**
**Total muscle mass kg)**	13.88 (12.19, 15.18)	13.98 (13.24, 15.26)	>0.5	21.22±1.75	21.95±2.51	0.234
**Arm fat mass (kg)**	3.16 (2.38, 3.62)	3.21 (2.63, 3.78)	>0.5	2.51 (1.76, 2.91)	2.97 (2.60, 3.49)	**0.008**
**Leg fat mass (kg)**	9.12 (6.95, 12.01)	9.25 (7.98, 11.41)	>0.5	6.37 (5.27, 7.95)	7.25 (6.29, 9.73)	**0.042**
**Arm muscle mass (kg)**	3.36±0.65	3.49±0.62	0.469	5.65 (5.14, 6.32)	6.19 (5.56, 6.35)	0.083
**Leg muscle mass (kg)**	10.83±1.65	10.96±1.62	>0.5	15.80±1.82	16.56±2.69	0.250
**6MW**	304 ± 129	310 ± 108	>0.5	364 (285, 413)	340 (277, 400)	>0.5
**SPPB**	8 (6, 9)	7 (6, 8.5)	0.440	8 (6, 8)	8 (6, 9)	0.220
**Grip strength (kg)**	13.69 ± 3.76	13.41 ± 2.99	>0.5	24.78 ± 6.61	23.29 ± 3.81	0.319
**QMVC (kg)**	12.20 (7.15, 15.30)	10.20 (8.70, 14.40)	>0.5	17.30 (12.45, 20.88)	15.40 (13.08, 21.50)	>0.5

BMI: body mass index, SARC-F: Strength, assistance with walking, rising from a chair, climbing stairs, and falls questionnaire score, SPPB: Short Physical Performance Battery score: QMVC: Quadriceps Maximal Voluntary contraction. For normally distributed data values are given as mean ± SD and for data that did not show a normal distribution values are median (interquartile range). Body composition measurements were not available for 1 male. QMVC measurements were not available for 1 female and 6 males. 6MW distance was not available for 1 male.

**Fig 2:**
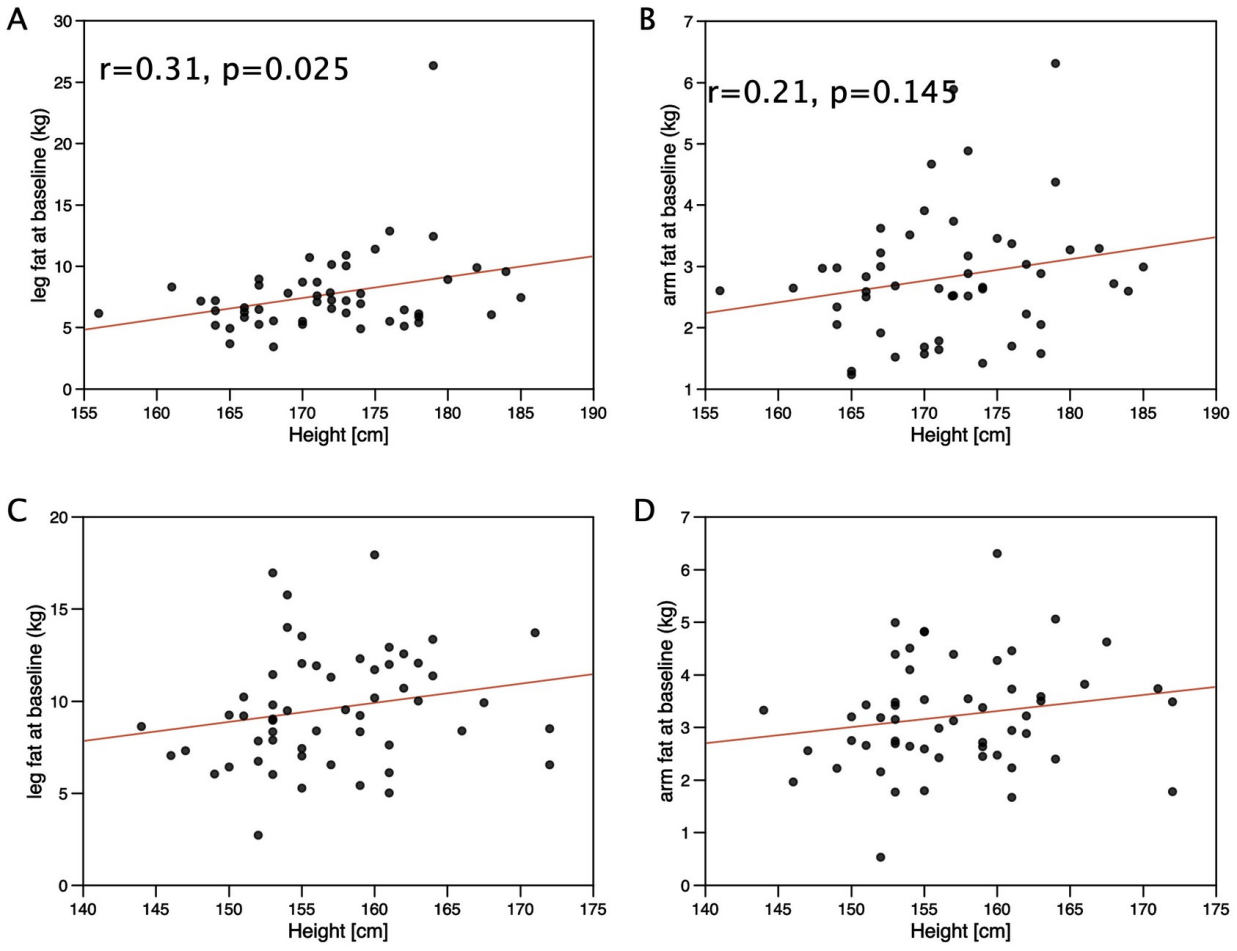
Association of height with limb fat mass in individuals in the LACE study. Height was compared with limb fat mass in males and females from the LACE study. In males there was a weak association of leg fat mass with height (r = 0.31, p = 0.025) but not in females. Arm fat mass was not associated with height in males or females.

**Fig 3:**
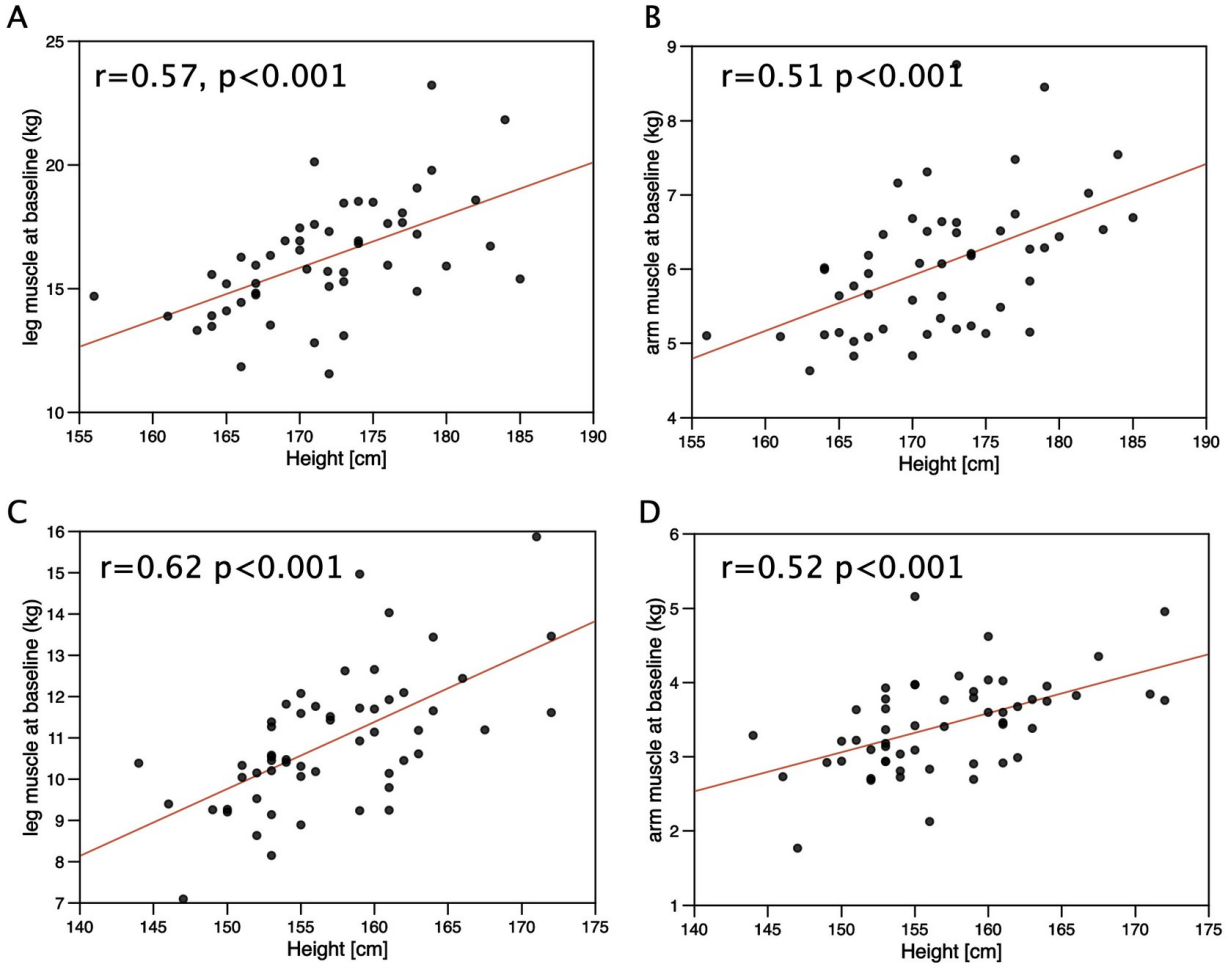
Association of height with limb muscle mass in individuals in the LACE study. Height was compared with limb muscle mass in males and females from the LACE study. In both males (r = 0.57 p<0.001) and females (r = 0.61, p<0.001) there was a strong association of leg muscle mass with height. Arm muscle mass was also associated with height in both males (r = 0.51, p<0.001) and females (r = 0.52, p<0.001).

Given the difference in height between the genotypes, we investigated the associations of limb muscle and fat mass with height. In males, leg and limb fat mass were weakly associated with height (r = 0.31, p = 0.025 and r = 0.30, p = 0.032 respectively) but there was no significant association between arm fat mass and height (r = 0.21, p = 0.145) nor were leg or arm fat associated with height in females (Fig 2). Height was directly proportional to leg muscle mass and arm muscle mass in both males (height vs leg muscle mass, r = 0.57, p<0.001, vs arm muscle mass r = 0.51, p<0.001, Fig 3A and 3B) and females (height vs leg muscle mass, r = 0.62, p<0.001, vs arm muscle mass r = 0.52, p<0.001, Fig 3C and 3D) but there was no association between height and either grip or leg strength in either sex.

After correction for height, arm fat mass remained significantly higher in AA/AG males than in GG males (mean_adj_ = 2.98kg (95%CI 2.60–3.41) vs 2.33kg (95% CI 2.01–2.69) respectively, p = 0.019, Table 3). While mean adjusted leg fat mass remained higher in AA/AG males than GG males this did not reach statistical significance (Table 3).

**Table 3:** Arm and leg fat mass in LACE males adjusted for height.

	**rs10783846 (males)**		
**Adjusted values**	**GG**	**AA/AG**	**P value**
**Arm fat mass_Adj_ (kg)**	2.33 (95%CI 2.01–2.69)	2.98 (95%CI 2.60–3.41)	**.019**
**Leg fat mass_Adj_ (kg)**	6.65 (95%CI 5.81–7.61)	7.76 (95%CI 6.84–8.80)	0.109
	**rs2854464 (males)**		
	**AA**	**AG/GG**	**P value**
**Arm fat mass_Adj_ (kg)**	2.39 (95%CI 2.07–2.75)	2.95 (95%CI 2.56–3.41)	**0.045**
**Leg fat mass_Adj_ (kg)**	6.91 (95%CI 6.06–7.89)	7.54 (95%CI 6.59–8.63)	0.366

Limb fat mass was log_2_ transformed and corrected for height using the General Linear Model in SPSS, mean values and 95% CI were calculated in SPSS. Values presented were calculated as 2^log corrected^. See S2 File for summary statistical output.

### rs2854464

**Fig 4:**
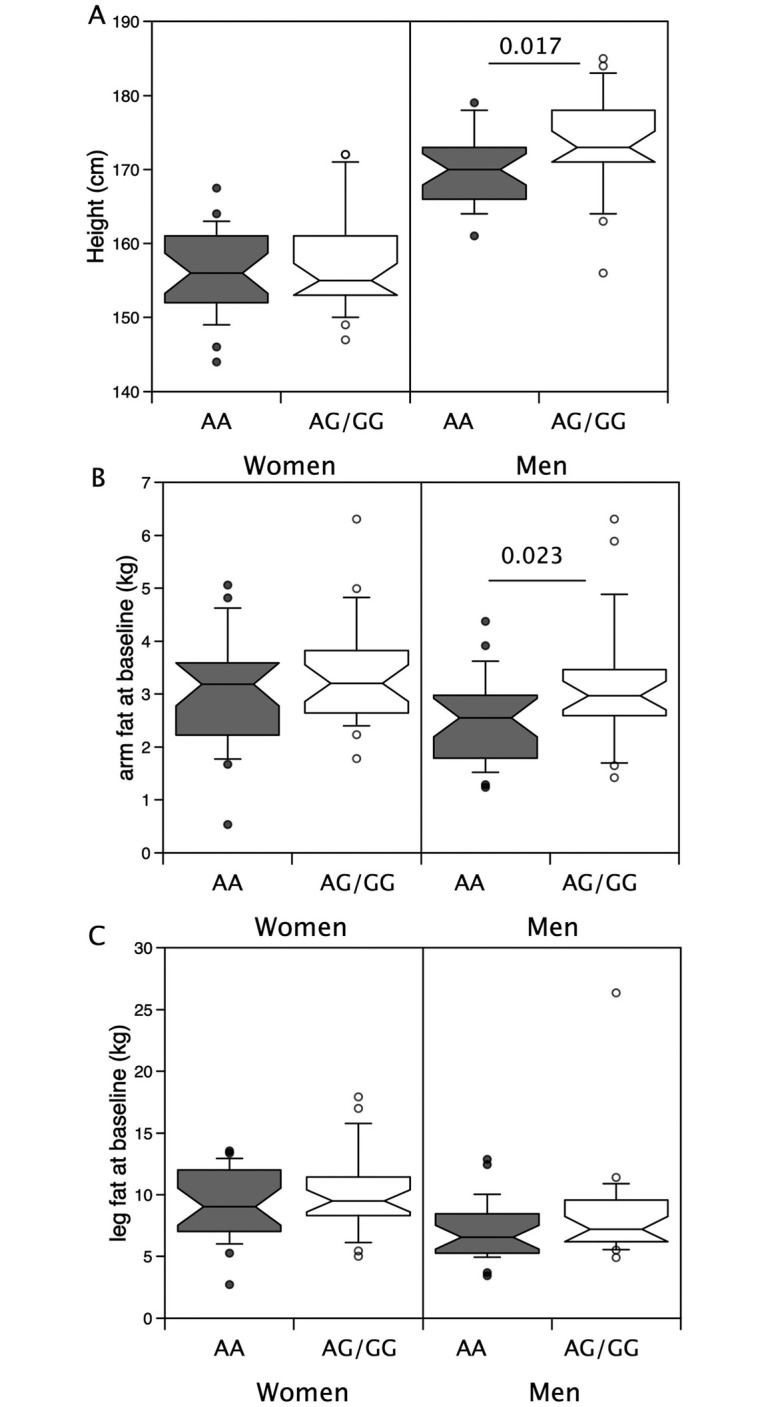
Associations of rs2854464 with height and limb fat mass in males and females in the LACE study. Height and limb fat were compared in males and females possessing the minor allele for rs2854464 with those homozygous for the major allele. Median height (A, p = 0.023), arm fat mass (B, p = 0.008) and leg fat mass (C, p = 0.046) were higher in males with the minor allele than their counterparts homozygous for the major allele. However, in females there were no differences.

Analysis of the rs2854464 allele showed a similar pattern to that described for rs10783486 both with no associations with total muscle mass or limb muscle mass (Table 4 and S2 Fig) but with the males carrying the minor allele (rs2854464 AG/GG) being taller than males homozygous for the major allele (rs2854464 AG/GG: 174±7 cm vs AA: 170±5cm, p = 0.017, Fig 4A). AG/GG males also had higher arm fat mass (median = 2.97kg (2.60, 3.46) vs 2.55kg (1.82, 2.95)), but although median leg fat mass was higher in AG/GG individuals this did not reach statistical significance (Fig 4B and 4C). Again, the difference in arm fat mass was retained after correction for height (AG/GG, mean_adj_ 2.95kg (95%CI 2.56–3.41) vs GG, mean_adj_ 2.39kg (95%CI 2.07–2.75), p = 0.045, Table 3). No differences were seen in females analysed as a group alone.

**Table 4:** Effect of the minor allele of rs2854464 on body composition and strength in females and males at baseline.

	rs2854464					
	female			male		
	AA	AG/GG	P value	AA	AG/GG	P value
**n**	27	29		27	25	
**Weight**	62.3±10.9	66.0±10.6	0.194	80.2 (74.2, 86.0)	85.3 (77.2, 96.0)	0.107
**BMI (kg/m^2^)**	25.4±3.6	26.6±3.8	0.286	27.7 (25.5, 30.1)	28.3 (25.5, 31.4)	>0.5
**Height (m)**	156 (152, 161)	155 (153, 161)	>0.5	170±5	174±7	**0.017**
**Total muscle mass (kg)**	13.87 (12.37, 15.23)	13.98 (13.32, 15.45)	0.441	21.41±1.80	21.81±2.56	>0.5
**Arm fat mass (kg)**	3.19 (2.33, 3.56)	3.21 (2.64, 3.82)	>0.5	2.55 (1.82, 2.95)	2.97 (2.60, 3.46)	**0.023**
**Leg fat mass (kg)**	9.16 ± 2.82	10.05 ± 3.27	0.308	6.55 (5.31, 8.40)	7.18 (6.20, 9.58)	0.158
**Arm muscle mass (kg)**	3.42 ± 0.64	3.42 ± 0.63	>0.5	5.72 (5.15, 6.42)	6.18 (5.49, 6.64)	0.132
**Leg muscle mass (kg)**	10.83 ± 1.63	10.88 ± 1.68	>0.5	15.85 ± 1.88	16.57 ± 2.72	0.271
**6MW**	322 (240, 384)	308 (258, 350)	0.496	372 (293, 414)	325 (266, 400)	0.360
**SPPB**	8 (6, 10)	7 (6, 8)	0.298	8 (6, 8)	8 (6, 9)	>0.5
**Grip strength (kg)**	14.4 (11.8, 16.0)	12.7 (11.3, 16.7)	0.422	24.6 ± 6.3	23.4 ± 4.2	0.425
**QMVC (kg)**	12.4 ± 6.0	10.6 ± 4.0	0.217	15.6 (12.3, 19.9)	17.4 (13.5, 22.3)	0.322

BMI: body mass index, SARC-F: Strength, assistance with walking, rising from a chair, climbing stairs, and falls questionnaire score, SPPB: Short Physical Performance Battery score: QMVC: Quadriceps Maximal Voluntary contraction. For normally distributed data values are given as mean ± SD and for data that did not show a normal distribution values are median (interquartile range). Body composition measurements were not available for 1 male. QMVC measurements were not available for 1 female and 6 males. 6MW distance was not available for 1 male.

In the UK biobank neither polymorphism associated with height or with arm fat mass at a significance that exceeded p = 10^−5^ (S3 Fig). Similarly in the GIANT consortium analysis as a whole there was no association of either polymorphism with height. Limiting the GIANT consortium data set to individuals of European ancestry increased the likelihood of these polymorphisms associating with height (p = 2.7*10^−5^) but did not reach the threshold of 10^−8^ usually used in GWAS studies to suggest significance. These analyses were not however, separated by sex or by age so it is not possible to directly compare the analyses. Widening the search of the GIANT European subset to polymorphisms within the ACVR1B locus (Chromosome 12: 52,345,451–52,390,862 using GRCh37.p13) identified rs10783484 (at Chromosome 12: 52331681), and a small cluster of polymorphisms from rs10747626 at Chromosome 12:52460424 to rs11521 at Chromosome 12:52471159 that show stronger associations (p = 10^−6^–10^−7^) with height in individuals of European ancestry.

### Expression of different alleles in skeletal muscle

Previous data have suggested that the rs2854464 polymorphism is located in a miR-24 binding site and that the major A allele has greater affinity for miR-24 leading to a reduction in ACVR1B mRNA. If this hypothesis was correct, it might be expected that there would be differential expression of the two ACVR1B alleles with the minor allele being expressed at higher levels. We therefore examined the GTEx dataset for single tissue eQTLs for rs2854464. Consistent with the hypothesis the expression of the minor allele was higher in skeletal muscle than expression of the major allele (S4 Fig). In a multi-tissue comparison, expression of the minor allele was also higher in the lung, heart, and regions of the central nervous system (S5 Fig). rs10783486 showed a similar pattern with higher expression of the minor allele. Given the linkage of these two alleles, a shared relative expression is expected.

## Discussion

These data show that males in the LACE cohort with at least one minor allele for either rs10783486 or rs2854464 were taller than those homozygous for the major allele and had more arm fat but we did not see any associations of these alleles with body composition in females. Previous studies have shown that polymorphisms in *ACVR1B* associate with physical strength and with muscle mass [21–23]. We were not able to replicate these findings in our cohort of individuals with sarcopenia even though the alleles were in Hardy Weinberg equilibrium within our sample set, with allele frequencies similar to those published for athletic populations and for the general population [21–23]. These observations may be a consequence of the size of the effect of the polymorphisms in the context of age-related wasting and our small sample size. Thus, confirmation of the findings both positive and negative in a much larger study is required. It is also possible that the lack of association with muscle mass and strength are a consequence of the selection process itself. All individuals in the study were selected based on being sarcopenic. As muscle mass and strength are components of the diagnosis and we did not have a control group with normal muscle mass or strength, any association between an SNP and muscle strength may be lost as a consequence of collider bias [33]. The similarity between the allele frequencies in our study and the populations where an association has been observed reduces the likelihood of this possibility. We did find that the alleles previously associated with increased muscle strength in the majority of studies reported to date, were associated with a smaller limb fat compartment measured by DXA in male individuals with sarcopenia, consistent with these alleles associating with body composition.

Previous studies have shown that muscle strength measured as grip strength or leg strength are proportional to height [34–36]. It is perhaps surprising therefore that males with the minor alleles were taller than those who were homozygous for the major alleles given that minor alleles have previously been associated with higher muscle mass. However, in our cohort whilst height is positively associated with both arm and leg muscle mass, it was not associated with grip or quadriceps strength. It is also interesting to note that in the GELAK cohort, the presence of the minor allele was enriched in athletes participating in team sports who were also the tallest group [25].

The associations that we identify with height were not identified in the previous studies of these alleles and are not apparent in the GIANT consortium at p<10^−8^. However, SNPs in the ACVR1B locus were associated with height in their recent study [37]. Furthermore, SNPs in loci containing other important components of the activin/myostatin signalling system were also associated with height including both ACVR2A and ACVR2B, as well as SMAD3, SMAD6 and SMAD7. Other studies support a role for ACVR1B signalling in the determination of body height. For examples exonic polymorphisms in *MAGI2*, a protein involved in the assembly of the activin receptor complex, have been shown to associate with early childhood height in Vietnamese and Korean children [38]. In animals, polymorphisms in myostatin have been associated with height as well as with increased muscle mass [39]. However, it is also possible that the association we see is due to linkage with adjacent loci as we identified stronger associations with polymorphic variants in a region encompassing NRF4A1 and ATG101.

Although our data cannot demonstrate causality, they raise the possibility that the accumulation of extramuscular limb fat may be influenced by activin receptor signalling—a hypothesis consistent with known effects of both myostatin and activin. Previous studies have shown that activin A signalling increases the proliferation of adipocyte progenitors but reduces adipocyte differentiation [40, 41] and myostatin increases the commitment of pluripotent mesenchymal cells to the adipocyte lineage and away from the myogenic lineage [28, 29]. By increasing the pool of adipocyte progenitors therefore, increased activin signalling may increase fat mass over time in an analogous manner to the effect of bone morphogenetic proteins (BMPs) on muscle formation and hypertrophy. In myoblasts, BMP signalling promotes proliferation but inhibits differentiation [42, 43]. The consequent increase in the pool of cells capable of differentiation into muscle leads to hypertrophy. Our data (increased extramuscular fat in individuals carrying the minor alleles) would be consistent with the minor alleles associating with increased myostatin/activin signalling. This observation would also be consistent with the suggestion that in athletes and other non-sarcopenic individuals the minor allele associates with reduced muscle mass and most likely therefore increased myostatin/activin signalling. Mechanistically other studies have hypothesised that the rs2854464 polymorphism is located in a miR-24 binding site and that the major A allele has a greater affinity for miR-24 leading to a reduction in ACVR1B mRNA and targeting of ACVR1B has been demonstrated in erythrocytes [44]. Consistent with a role for this polymorphism in ACVR1B mRNA levels, GTEx eQTL data indicate that in human skeletal muscle expression of the minor allele is higher than expression of the major allele.

Small numbers in our analysis limit our ability to draw strong conclusions about differences between males and females, but it is possible that the difference in fat accumulation may be either more detectable in males due to differences in relative fat proportions, or may be larger in males than in females. It is possible that the increase in fat is a consequence of reduced relative strength and activity, or is due to a difference in muscle metabolism that contributes to the difference in strength but leads to a greater resistance to insulin over time and therefore an increase in the accumulation of fat that accompanies this. However, even though the median strength of the rs10783486 GG individuals was higher than that of the AA/AG individuals, consistent with the known associations of this polymorphism [21], we did not identify any significant differences in strength or muscle mass in this cohort. It is therefore less likely that the increase in fat is a consequence of such changes in muscle mass or metabolism. It is also possible that the difference is a consequence of a tendency towards adipocyte formation, proliferation or hypertrophy in the presence of higher activin receptor signalling. This effect of activin, coupled to any effect of myostatin altering the balance of differentiation towards adipocytes, is consistent with the associations we observe.

The greater height in those carrying the minor allele is more difficult to rationalise if the minor alleles are associated with higher ACVR1B protein levels as both activin and myostatin inhibit bone growth [45, 46]. However, this interpretation assumes that the effect of ACVR1B signalling on height is direct rather than an indirect effect on other systems controlling growth and development or through linkage with other genes in the same region.

### Limitations of the study

This analysis is limited by the size of the study populations and by the lack of a control group of individuals of similar age who did not have sarcopenia. Both these limitations arise from the samples coming from the LACE clinical trial. Firstly, the trial was placebo-controlled trial within the sarcopenia population, as a result there was no recruitment of individuals without sarcopenia. Secondly, recruitment of individuals to the trial proved difficult [47] leading the sponsor to stop recruitment, thereby restricting the number of samples available. The results therefore require verification in larger studies. The study differs from other studies of these alleles by selecting individuals who are already sarcopenic. Whilst the information is therefore restricted to this demographic and may not be transferable to other groups of individuals, the population studied is relevant to finding treatments in the clinical setting where individuals present with sarcopenia.

Our analysis of total muscle mass is limited by being derived from bioimpedance rather than DXA. The bioimpedance was conducted as a screening tool to limit the number of DXA scans and the analysis of the DXA data was carried out to identify changes in appendicular muscle mass specifically. However, within our data set the bioimpedance measurements of total muscle mass are tightly correlated with the limb muscle measurements [(r = 0.815, p<0.001 arm and r = 0.830, p<0.001 leg muscle), associations that are similar to the association between arm and leg muscle measurements, both by DXA (r = 0.864, p<0.001 arm vs leg muscle)]. As we did not see any differences in muscle mass based on DXA measurements, it is unlikely that the lower reliability of the bioimpedance measurements affected the outcome.

A further limitation is that, as a cross-sectional observational study, data demonstrate association only, and not causation. Indeed, it is not currently clear whether the changes in receptor mRNA observed in the GTEx are present in all tissues and are sufficient to significantly modify activin receptor signalling.

## Conclusions

Our data suggest that polymorphisms in the activin I B receptor locus are associated with height and limb fat mass rather than muscle mass and strength in older men with sarcopenia. These findings are potentially of clinical significance; interventions that target the activin/myostatin pathway could potentially exert beneficial effects on extramuscular fat, at least in the limbs, with consequent amelioration of the deleterious effects of such fat on skeletal muscle physiology. Such mechanisms may be of particular interest in improving muscle function in individuals who have both obesity and sarcopenia (‘sarcopenic obesity’). If functional studies validate our findings, the obese and sarcopenic population would be a key target for future intervention studies using myostatin/activin pathway modulators.

## Supporting information

S1 File All data used. (XLSX)

S2 File SPSS statistics output from ANCOV analysis. (XLSX)

S1 Table Genotype frequencies in sarcopenia. (DOCX)

S1 Fig Associations of rs10783846 with limb muscle mass in individuals in the LACE study. Arm and leg muscle masses were compared in males and females possessing the minor allele for rs10783846 with those homozygous for the major allele. Median arm muscle mass and leg muscle mass did not differ based on possession of the minor allele of rs10783846 in either gender.
(TIF)

S2 Fig Associations of rs2854464 with limb muscle mass in individuals in the LACE study. Arm and leg muscle masses were compared in males and females possessing the minor allele for rs2854464 with those homozygous for the major allele. Median arm muscle mass and leg muscle mass did not differ based on possession of the minor allele of rs2854464in either gender.
(TIF)

S3 Fig Association of polymorphisms in the ACVR1B locus with height and arm fat in the UK biobank study. The UK biobank data set (http://geneatlas.roslin.ed.ac.uk/) was investigated for associations between polymorphisms in the ACVR1B locus (+/- 50kbp) and either standing height (A) or left arm fat mass (B). No polymorphisms showed associations with significance p<10^−5^ with either physiological trait in the whole cohort.
(TIF)

S4 Fig eQTL analysis of rs2854464 and rs10783846 in skeletal muscle. The GTEx data set was analysed to determine whether either polymorphism showed differential expression. In skeletal muscle both minor alleles of rs2854464 and rs10783846 were more highly expressed than the major alleles. The data are shown in violin plot form taken from the GTEx portal.
(TIF)

S5 Fig Tissue eQTL analysis of rs2854464. The GTEx data set was analysed for eQTL associations with rs2854464. The strongest effects of the polymorphism on expression were observed in the lung and in skeletal muscle.
(TIF)

S1 Checklist *PLOS ONE* clinical studies checklist. (DOCX)

S2 Checklist STROBE statement—Checklist of items that should be included in reports of observational studies. (DOCX)

## References

[1] Kemp PR, Griffiths M, Polkey MI. Muscle wasting in the presence of disease, why is it so variable? Biol Rev Camb Philos Soc. 2019;94(3):1038–55. doi: 10.1111/brv.12489 30588725

[2] von Haehling S, Morley JE, Anker SD. From muscle wasting to sarcopenia and myopenia: update 2012. J Cachexia Sarcopenia Muscle. 2012;3(4):213–7. doi: 10.1007/s13539-012-0089-z 23160774 PMC3505577

[3] Janssen I, Heymsfield SB, Ross R. Low relative skeletal muscle mass (sarcopenia) in older persons is associated with functional impairment and physical disability. J Am Geriatr Soc. 2002;50(5):889–96. doi: 10.1046/j.1532-5415.2002.50216.x 12028177

[4] Malmstrom TK, Miller DK, Simonsick EM, Ferrucci L, Morley JE. SARC-F: a symptom score to predict persons with sarcopenia at risk for poor functional outcomes. J Cachexia Sarcopenia Muscle. 2016;7(1):28–36. doi: 10.1002/jcsm.12048 27066316 PMC4799853

[5] Cruz-Jentoft AJ, Baeyens JP, Bauer JM, Boirie Y, Cederholm T, Landi F, et al. Sarcopenia: European consensus on definition and diagnosis: Report of the European Working Group on Sarcopenia in Older People. Age Ageing. 2010;39(4):412–23. doi: 10.1093/ageing/afq034 20392703 PMC2886201

[6] Cruz-Jentoft AJ, Bahat G, Bauer J, Boirie Y, Bruyere O, Cederholm T, et al. Sarcopenia: revised European consensus on definition and diagnosis. Age Ageing. 2019;48(1):16–31. doi: 10.1093/ageing/afy169 30312372 PMC6322506

[7] Metter EJ, Lynch N, Conwit R, Lindle R, Tobin J, Hurley B. Muscle quality and age: cross-sectional and longitudinal comparisons. J Gerontol A Biol Sci Med Sci. 1999;54(5):B207–18. doi: 10.1093/gerona/54.5.b207 10362000

[8] Naimo MA, Varanoske AN, Hughes JM, Pasiakos SM. Skeletal Muscle Quality: A Biomarker for Assessing Physical Performance Capabilities in Young Populations. Front Physiol. 2021;12:706699. doi: 10.3389/fphys.2021.706699 34421645 PMC8376973

[9] Faron A, Sprinkart AM, Kuetting DLR, Feisst A, Isaak A, Endler C, et al. Body composition analysis using CT and MRI: intra-individual intermodal comparison of muscle mass and myosteatosis. Sci Rep. 2020;10(1):11765. doi: 10.1038/s41598-020-68797-3 32678260 PMC7367311

[10] Peterson MD, Liu D, Gordish-Dressman H, Hubal MJ, Pistilli E, Angelopoulos TJ, et al. Adiposity attenuates muscle quality and the adaptive response to resistance exercise in non-obese, healthy adults. Int J Obes (Lond). 2011;35(8):1095–103. doi: 10.1038/ijo.2010.257 21139562 PMC4147945

[11] Rahemi H, Nigam N, Wakeling JM. The effect of intramuscular fat on skeletal muscle mechanics: implications for the elderly and obese. J R Soc Interface. 2015;12(109):20150365. doi: 10.1098/rsif.2015.0365 26156300 PMC4535407

[12] Maffiuletti NA, Jubeau M, Munzinger U, Bizzini M, Agosti F, De Col A, et al. Differences in quadriceps muscle strength and fatigue between lean and obese subjects. Eur J Appl Physiol. 2007;101(1):51–9. doi: 10.1007/s00421-007-0471-2 17476522

[13] Tomlinson DJ, Erskine RM, Morse CI, Winwood K, Onambele-Pearson GL. Combined effects of body composition and ageing on joint torque, muscle activation and co-contraction in sedentary women. Age (Dordr). 2014;36(3):9652. doi: 10.1007/s11357-014-9652-1 24744050 PMC4082607

[14] Tomlinson DJ, Erskine RM, Winwood K, Morse CI, Onambele GL. Obesity decreases both whole muscle and fascicle strength in young females but only exacerbates the aging-related whole muscle level asthenia. Physiol Rep. 2014;2(6). doi: 10.14814/phy2.12030 24963030 PMC4208641

[15] Zane AC, Reiter DA, Shardell M, Cameron D, Simonsick EM, Fishbein KW, et al. Muscle strength mediates the relationship between mitochondrial energetics and walking performance. Aging Cell. 2017;16(3):461–8. doi: 10.1111/acel.12568 28181388 PMC5418194

[16] Chen MM, Zhao YP, Zhao Y, Deng SL, Yu K. Regulation of Myostatin on the Growth and Development of Skeletal Muscle. Front Cell Dev Biol. 2021;9:785712. doi: 10.3389/fcell.2021.785712 35004684 PMC8740192

[17] Chen JL, Walton KL, Winbanks CE, Murphy KT, Thomson RE, Makanji Y, et al. Elevated expression of activins promotes muscle wasting and cachexia. FASEB J. 2014;28(4):1711–23. doi: 10.1096/fj.13-245894 24378873

[18] Klimentidis YC, Bea JW, Thompson P, Klimecki WT, Hu C, Wu G, et al. Genetic Variant in ACVR2B Is Associated with Lean Mass. Med Sci Sports Exerc. 2016;48(7):1270–5. doi: 10.1249/MSS.0000000000000889 26848890 PMC4911281

[19] Walsh S, Metter EJ, Ferrucci L, Roth SM. Activin-type II receptor B (ACVR2B) and follistatin haplotype associations with muscle mass and strength in humans. J Appl Physiol (1985). 2007;102(6):2142–8. doi: 10.1152/japplphysiol.01322.2006 17347381 PMC2646094

[20] Zhang X, Ye L, Li X, Chen Y, Jiang Y, Li W, et al. The association between sarcopenia susceptibility and polymorphisms of FTO, ACVR2B, and IRS1 in Tibetans. Mol Genet Genomic Med. 2021;9(8):e1747. doi: 10.1002/mgg3.1747 34302448 PMC8404241

[21] Windelinckx A, De Mars G, Huygens W, Peeters MW, Vincent B, Wijmenga C, et al. Comprehensive fine mapping of chr12q12-14 and follow-up replication identify activin receptor 1B (ACVR1B) as a muscle strength gene. Eur J Hum Genet. 2011;19(2):208–15. doi: 10.1038/ejhg.2010.173 21063444 PMC3025799

[22] Voisin S, Guilherme JP, Yan X, Pushkarev VP, Cieszczyk P, Massidda M, et al. ACVR1B rs2854464 Is Associated with Sprint/Power Athletic Status in a Large Cohort of Europeans but Not Brazilians. PLoS One. 2016;11(6):e0156316. doi: 10.1371/journal.pone.0156316 27253421 PMC4890799

[23] Khanal P, He L, Herbert AJ, Stebbings GK, Onambele-Pearson GL, Degens H, et al. The Association of Multiple Gene Variants with Ageing Skeletal Muscle Phenotypes in Elderly Women. Genes (Basel). 2020;11(12). doi: 10.3390/genes11121459 33291384 PMC7762041

[24] Thomaes T, Thomis M, Onkelinx S, Goetschalckx K, Fagard R, Lambrechts D, et al. Genetic predisposition scores associate with muscular strength, size, and trainability. Med Sci Sports Exerc. 2013;45(8):1451–9. doi: 10.1249/MSS.0b013e31828983f7 23439425

[25] Venckunas T, Degens H. Genetic polymorphisms of muscular fitness in young healthy men. PLoS One. 2022;17(9):e0275179. doi: 10.1371/journal.pone.0275179 36166425 PMC9514622

[26] Deng B, Zhang F, Wen J, Ye S, Wang L, Yang Y, et al. The function of myostatin in the regulation of fat mass in mammals. Nutr Metab (Lond). 2017;14:29. doi: 10.1186/s12986-017-0179-1 28344633 PMC5360019

[27] Zhu HJ, Pan H, Zhang XZ, Li NS, Wang LJ, Yang HB, et al. The effect of myostatin on proliferation and lipid accumulation in 3T3-L1 preadipocytes. J Mol Endocrinol. 2015;54(3):217–26. doi: 10.1530/JME-15-0038 25878062

[28] Feldman BJ, Streeper RS, Farese RV Jr., Yamamoto KR. Myostatin modulates adipogenesis to generate adipocytes with favorable metabolic effects. Proc Natl Acad Sci U S A. 2006;103(42):15675–80. doi: 10.1073/pnas.0607501103 17030820 PMC1592529

[29] Artaza JN, Bhasin S, Magee TR, Reisz-Porszasz S, Shen R, Groome NP, et al. Myostatin inhibits myogenesis and promotes adipogenesis in C3H 10T(1/2) mesenchymal multipotent cells. Endocrinology. 2005;146(8):3547–57. doi: 10.1210/en.2005-0362 15878958

[30] McPherron AC, Lee SJ. Suppression of body fat accumulation in myostatin-deficient mice. J Clin Invest. 2002;109(5):595–601. doi: 10.1172/JCI13562 11877467 PMC150888

[31] group Ls, Achison M, Adamson S, Akpan A, Aspray T, Avenell A, et al. Effect of perindopril or leucine on physical performance in older people with sarcopenia: the LACE randomized controlled trial. J Cachexia Sarcopenia Muscle. 2022. doi: 10.1002/jcsm.12934 35174663 PMC8977979

[32] Band MM, Sumukadas D, Struthers AD, Avenell A, Donnan PT, Kemp PR, et al. Leucine and ACE inhibitors as therapies for sarcopenia (LACE trial): study protocol for a randomised controlled trial. Trials. 2018;19(1):6. doi: 10.1186/s13063-017-2390-9 29301558 PMC5753568

[33] Munafo MR, Tilling K, Taylor AE, Evans DM, Davey Smith G. Collider scope: when selection bias can substantially influence observed associations. Int J Epidemiol. 2018;47(1):226–35. doi: 10.1093/ije/dyx206 29040562 PMC5837306

[34] Harbo T, Brincks J, Andersen H. Maximal isokinetic and isometric muscle strength of major muscle groups related to age, body mass, height, and sex in 178 healthy subjects. Eur J Appl Physiol. 2012;112(1):267–75. doi: 10.1007/s00421-011-1975-3 21537927

[35] Mendes J, Amaral TF, Borges N, Santos A, Padrao P, Moreira P, et al. Handgrip strength values of Portuguese older adults: a population based study. BMC Geriatr. 2017;17(1):191. doi: 10.1186/s12877-017-0590-5 28835211 PMC5569490

[36] Spruit MA, Sillen MJ, Groenen MT, Wouters EF, Franssen FM. New normative values for handgrip strength: results from the UK Biobank. J Am Med Dir Assoc. 2013;14(10):775 e5–11. doi: 10.1016/j.jamda.2013.06.013 23958225

[37] Yengo L, Vedantam S, Marouli E, Sidorenko J, Bartell E, Sakaue S, et al. A saturated map of common genetic variants associated with human height. Nature. 2022;610(7933):704–12. doi: 10.1038/s41586-022-05275-y 36224396 PMC9605867

[38] Kim HN, Lee EJ, Jung SC, Lee JY, Chung HW, Kim HL. Genetic variants that affect length/height in infancy/early childhood in Vietnamese-Korean families. J Hum Genet. 2010;55(10):681–90. doi: 10.1038/jhg.2010.88 20668459

[39] Bi Y, Feng B, Wang Z, Zhu H, Qu L, Lan X, et al. Myostatin (MSTN) Gene Indel Variation and Its Associations with Body Traits in Shaanbei White Cashmere Goat. Animals (Basel). 2020;10(1). doi: 10.3390/ani10010168 31963797 PMC7022945

[40] Zaragosi LE, Wdziekonski B, Villageois P, Keophiphath M, Maumus M, Tchkonia T, et al. Activin a plays a critical role in proliferation and differentiation of human adipose progenitors. Diabetes. 2010;59(10):2513–21. doi: 10.2337/db10-0013 20530742 PMC3279533

[41] Dani C. Activins in adipogenesis and obesity. Int J Obes (Lond). 2013;37(2):163–6. doi: 10.1038/ijo.2012.28 22370854

[42] Friedrichs M, Wirsdoerfer F, Flohe SB, Schneider S, Wuelling M, Vortkamp A. BMP signaling balances proliferation and differentiation of muscle satellite cell descendants. BMC Cell Biol. 2011;12:26. doi: 10.1186/1471-2121-12-26 21645366 PMC3149017

[43] Sartori R, Schirwis E, Blaauw B, Bortolanza S, Zhao J, Enzo E, et al. BMP signaling controls muscle mass. Nat Genet. 2013;45(11):1309–18. doi: 10.1038/ng.2772 24076600

[44] Wang Q, Huang Z, Xue H, Jin C, Ju XL, Han JD, et al. MicroRNA miR-24 inhibits erythropoiesis by targeting activin type I receptor ALK4. Blood. 2008;111(2):588–95. doi: 10.1182/blood-2007-05-092718 17906079

[45] Suh J, Kim NK, Lee SH, Eom JH, Lee Y, Park JC, et al. GDF11 promotes osteogenesis as opposed to MSTN, and follistatin, a MSTN/GDF11 inhibitor, increases muscle mass but weakens bone. Proc Natl Acad Sci U S A. 2020;117(9):4910–20. doi: 10.1073/pnas.1916034117 32071240 PMC7060712

[46] Chantry AD, Heath D, Mulivor AW, Pearsall S, Baud’huin M, Coulton L, et al. Inhibiting activin-A signaling stimulates bone formation and prevents cancer-induced bone destruction in vivo. J Bone Miner Res. 2010;25(12):2633–46. doi: 10.1002/jbmr.142 20533325

[47] Witham MD, Achison M, Aspray TJ, Avenell A, Band MM, Donnan PT, et al. Recruitment strategies for sarcopenia trials: lessons from the LACE randomized controlled trial. JCSM rapid communications. 2021;4(2):93–102.

