# Supplementary material for: Activin type I receptor polymorphisms and body composition in older individuals with sarcopenia—Analyses from the LACE randomised controlled trial
Source: PLoS One. 2023 Nov 14;18(11):e0294330. doi: 10.1371/journal.pone.0294330 (PMC10645316; doi:10.1371/journal.pone.0294330)
Supplement: S1 Table — (DOCX) [file pone.0294330.s003.docx]

**Table S1**

Genotype frequencies in sarcopenia

| rs10783486 | | | |
| --- | --- | --- | --- |
| GG | GA | AA | MAF |
| 49 | 51 | 7 | 0.30 |
| rs2854464 | | | |
| GG | GA | AA | MAF |
| 6 | 48 | 53 | 0.28 |
