# Supplementary figures and images for: Activin type I receptor polymorphisms and body composition in older individuals with sarcopenia—Analyses from the LACE randomised controlled trial
Source: PLoS One. 2023 Nov 14;18(11):e0294330. doi: 10.1371/journal.pone.0294330 (PMC10645316; doi:10.1371/journal.pone.0294330)

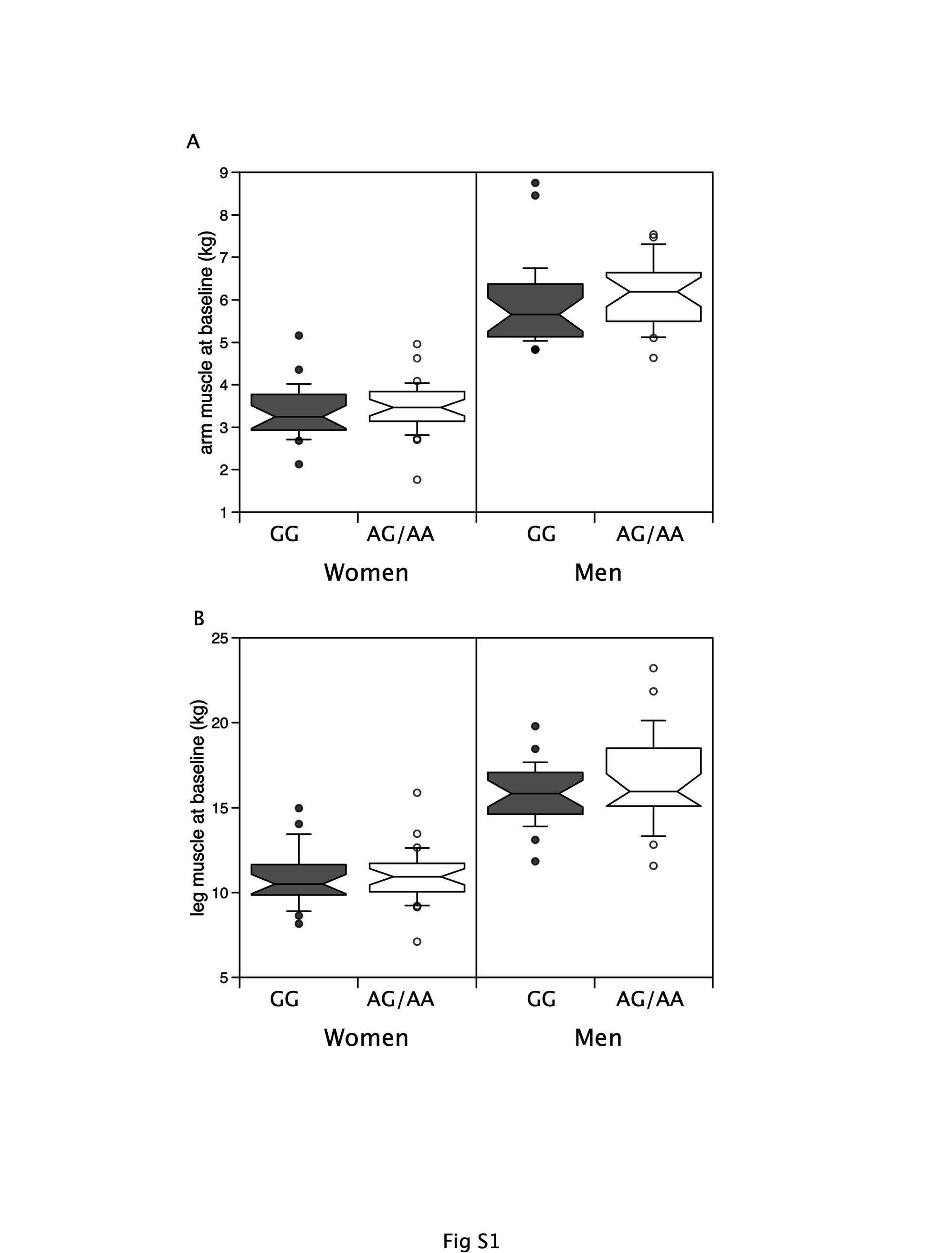

Supplement: S1 Fig — Arm and leg muscle masses were compared in males and females possessing the minor allele for rs10783846 with those homozygous for the major allele. Median arm muscle mass and leg muscle mass did not differ based on possession of the minor allele of rs10783846 in either gender. (TIF) [file pone.0294330.s004.tif]

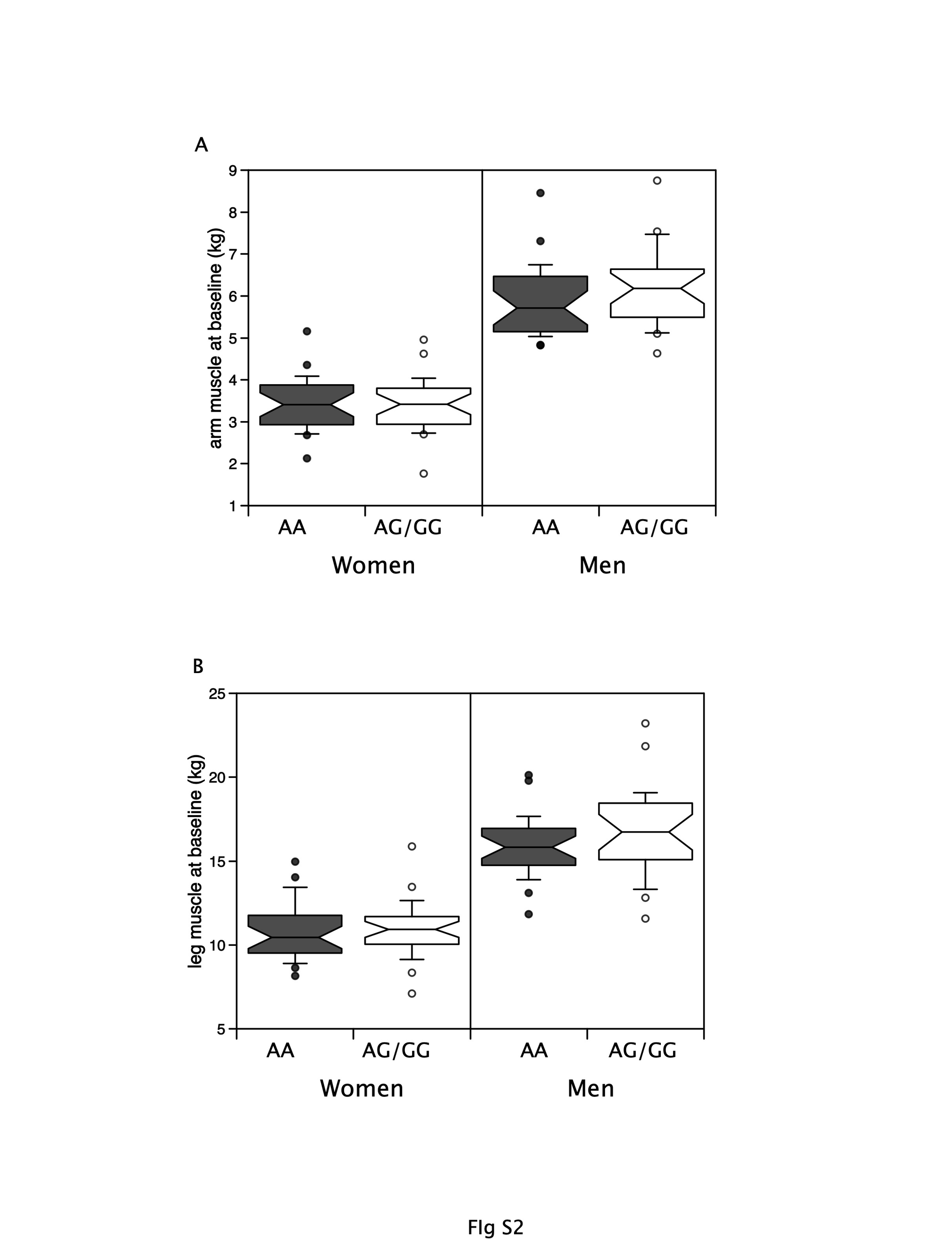

Supplement: S2 Fig — Arm and leg muscle masses were compared in males and females possessing the minor allele for rs2854464 with those homozygous for the major allele. Median arm muscle mass and leg muscle mass did not differ based on possession of the minor allele of rs2854464in either gender. (TIF) [file pone.0294330.s005.tif]

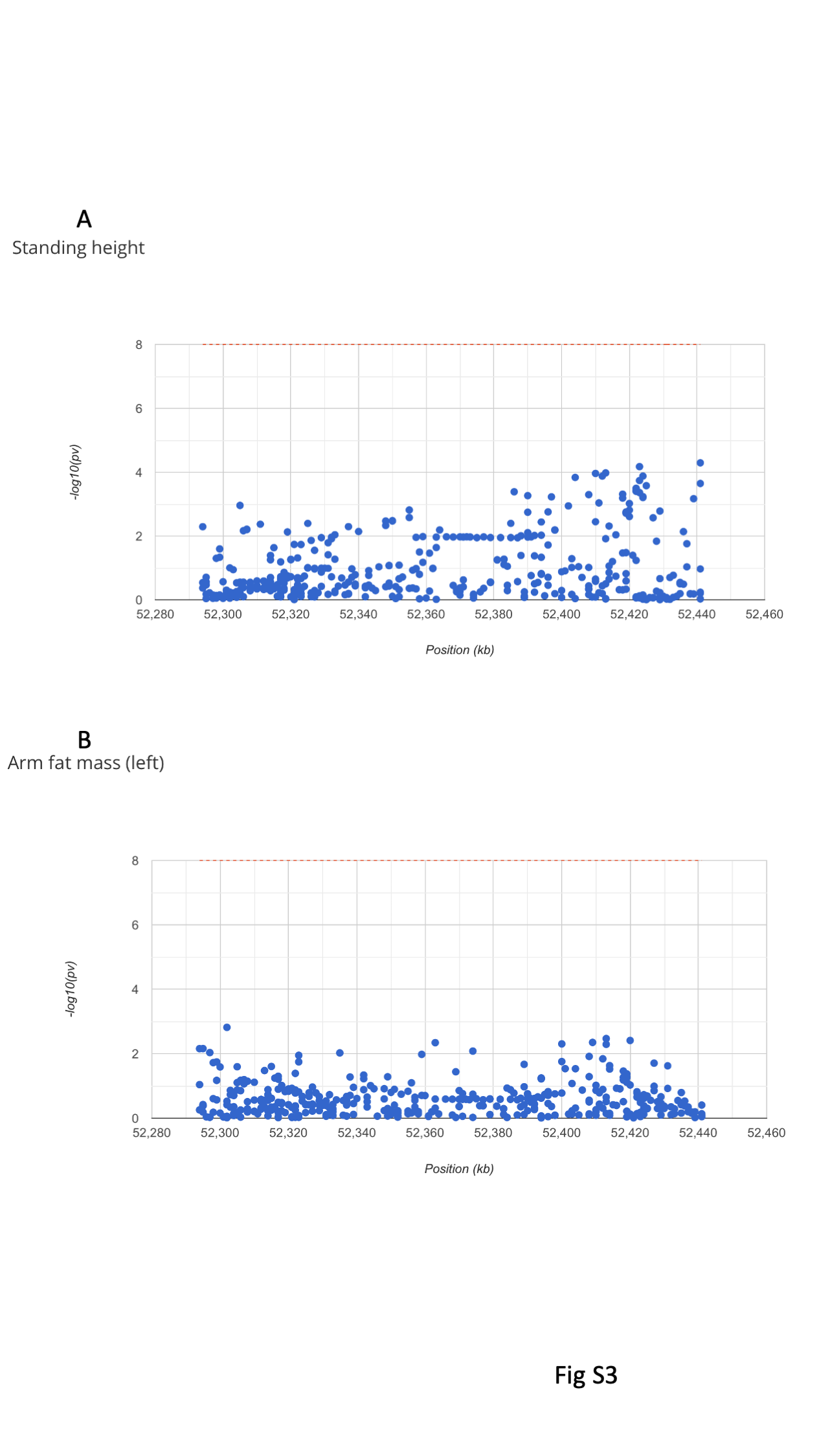

Supplement: S3 Fig — The UK biobank data set (http://geneatlas.roslin.ed.ac.uk/) was investigated for associations between polymorphisms in the ACVR1B locus (+/- 50kbp) and either standing height (A) or left arm fat mass (B). No polymorphisms showed associations with significance p<10−5 with either physiological trait in the whole cohort. (TIF) [file pone.0294330.s006.tif]

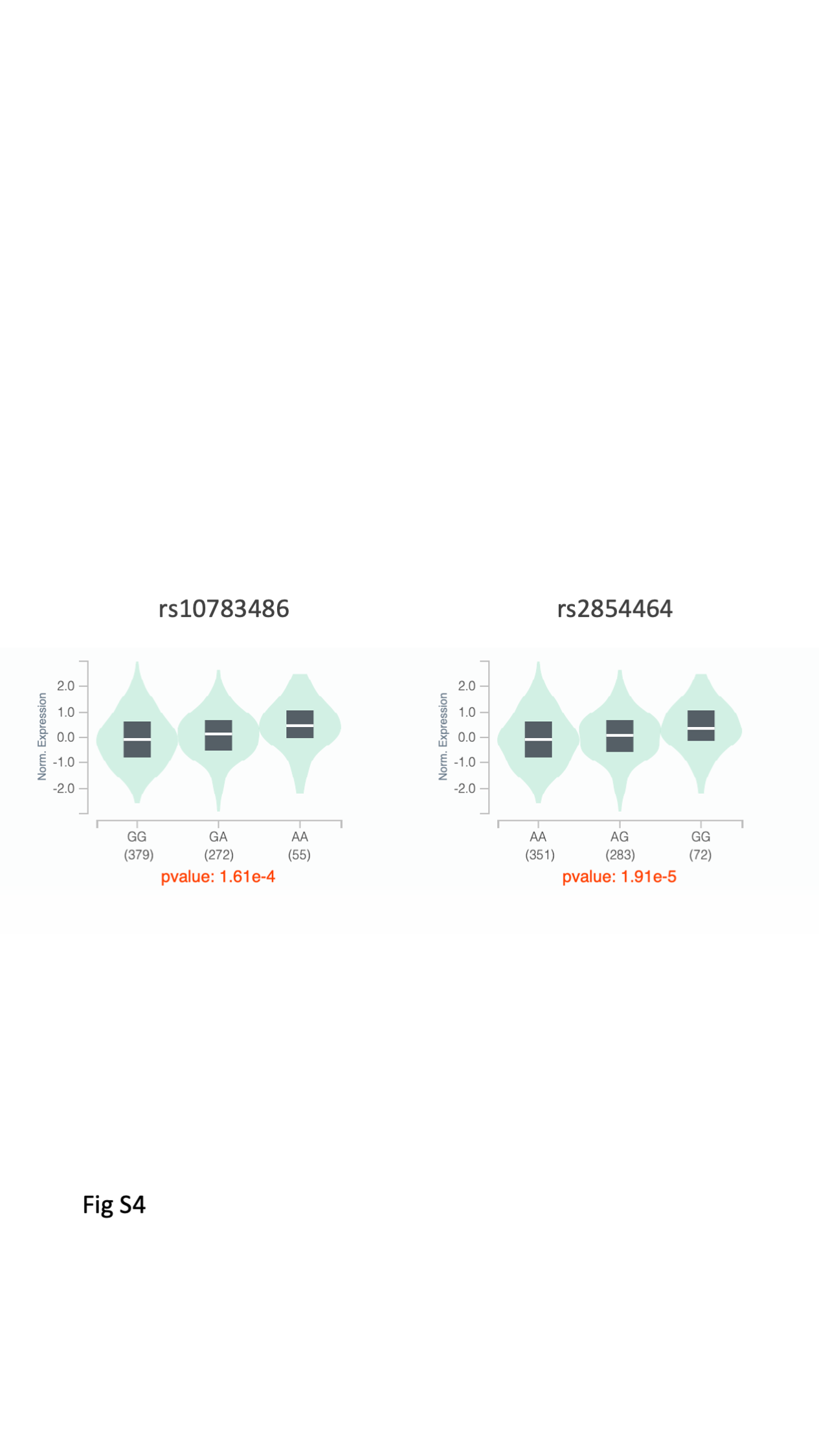

Supplement: S4 Fig — The GTEx data set was analysed to determine whether either polymorphism showed differential expression. In skeletal muscle both minor alleles of rs2854464 and rs10783846 were more highly expressed than the major alleles. The data are shown in violin plot form taken from the GTEx portal. (TIF) [file pone.0294330.s007.tif]

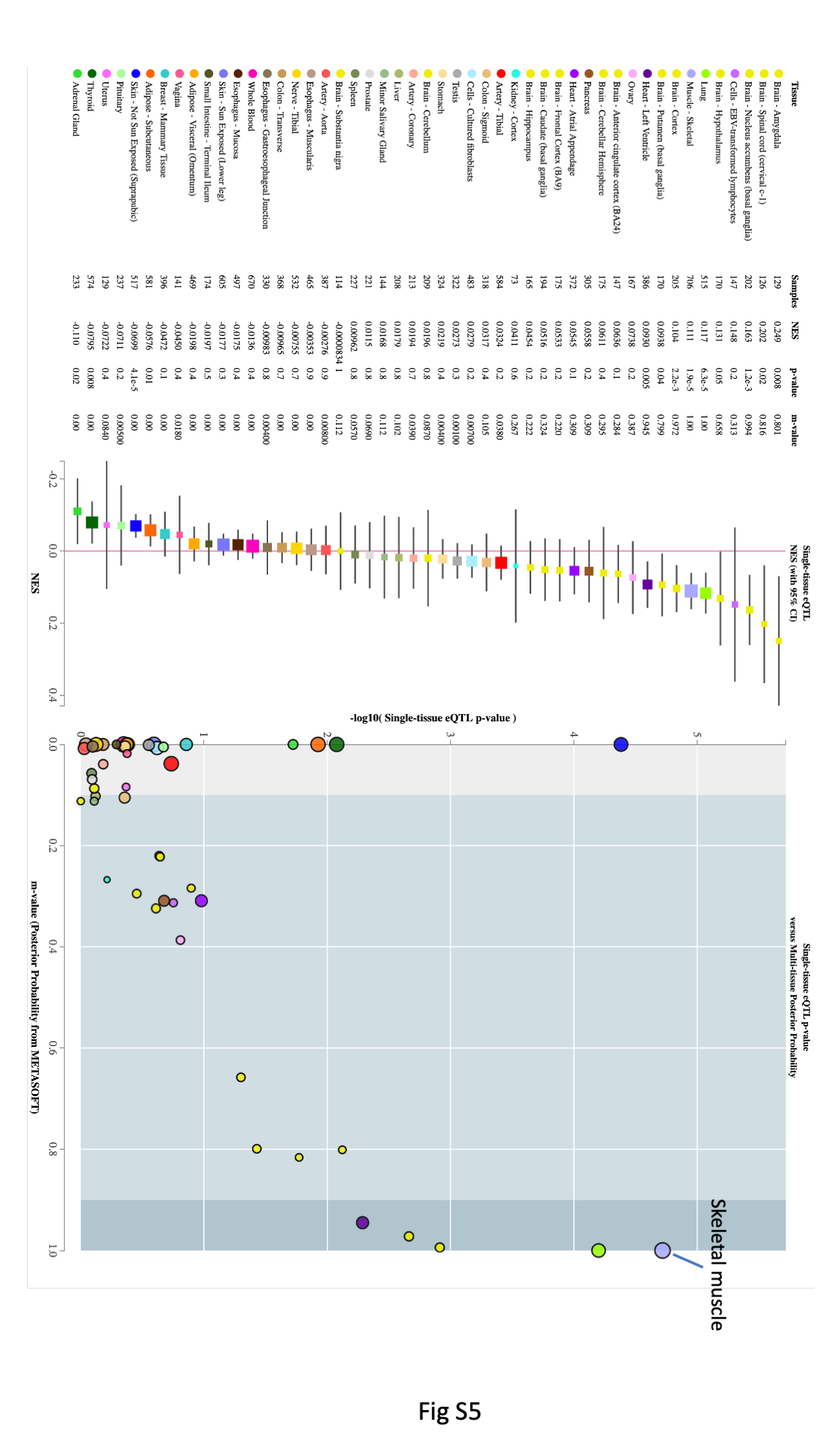

Supplement: S5 Fig — The GTEx data set was analysed for eQTL associations with rs2854464. The strongest effects of the polymorphism on expression were observed in the lung and in skeletal muscle. (TIF) [file pone.0294330.s008.tif]
